# Impact of coronavirus disease 2019-related clinic closures on HIV incidence in young adult MSM and transgender women in Kenya

**DOI:** 10.1097/QAD.0000000000003782

**Published:** 2023-11-27

**Authors:** Elizabeth Wahome, Fredrick O. Otieno, Joshua Kimani, Anders Boyd, Duncan Okall, Joseph Nzioka, Evans Gichuru, Elise van der Elst, Supriya D. Mehta, Robert C. Bailey, Susan M. Graham, Eduard J. Sanders

**Affiliations:** a KEMRI/Wellcome Trust Research Programme, Kilifi; b Nyanza Reproductive Health Society, Kisumu; c Sex Worker Outreach Program (SWOP), and Partners for Health and Development in Africa (PHDA), Nairobi, Kenya; d Public Health Service of Amsterdam, Department of Infectious Diseases; e Stichting HIV Monitoring; f Amsterdam UMC location University of Amsterdam, Infectious Diseases; g Department of Global Health, University of Amsterdam, Amsterdam, Netherlands; h University of Illinois at Chicago, IL; i University of Washington, Seattle, WA, USA; j University of Oxford, Headington, UK; k The Aurum Institute, Johannesburg, South Africa.

**Keywords:** coronavirus disease 2019, HIV incidence, Kenya, MSM, preexposure prophylaxis, transgender women

## Abstract

**Introduction:**

Little is known about the impact that the COVID-19 pandemic had on risk of HIV acquisition in sub-Saharan Africa. We assessed the impact of COVID-19-related clinic closures on HIV incidence in a cohort of gay, bisexual, and other men who have sex with men (MSM) and transgender women in Kenya.

**Methods:**

MSM and transgender women enrolled in a prospective, multicentre cohort study were followed quarterly for HIV testing, behaviour assessments, and risk. We estimated the HIV incidence rate and its 95% credible intervals (CrI) among participants who were HIV-negative before COVID-19-related clinic closure, comparing incidence rate and risk factors associated with HIV acquisition before vs. after clinic reopening, using a Bayesian Poisson model with weakly informative priors.

**Results:**

A total of 690 (87%) participants returned for follow-up after clinic reopening (total person-years 664.3 during clinic closure and 1013.3 after clinic reopening). HIV incidence rate declined from 2.05/100 person-years (95% CrI = 1.22–3.26, *n* = 14) during clinic closures to 0.96/100 person-years (95% CrI = 0.41–2.07, *n* = 10) after clinic reopening (IRR = 0.47, 95% CrI = 0.20–1.01). The proportion of participants reporting hazardous alcohol use and several sexual risk behaviours was higher during clinic closures than after clinic reopening. In multivariable analysis adjusting for study site and participant characteristics, HIV incidence was lower after clinic reopening (IRR 0.57, 95% CrI = 0.23–1.33). Independent risk factors for HIV acquisition included receptive anal intercourse (IRR 1.94, 95% CrI = 0.88–4.80) and perceived risk of HIV (IRR 3.03, 95% CRI = 1.40–6.24).

**Conclusion:**

HIV incidence during COVID-19-related clinic closures was moderately increased and reduced after COVID-19 restrictions were eased. Ensuring access to services for key populations is important during public health emergencies.

## Introduction

The emergence of the severe acute respiratory syndrome coronavirus 2 (SARS-CoV-2), the virus that causes coronavirus disease 2019 (COVID-19), in Kenya since March 2020 [1] led to interruptions in HIV prevention and treatment services, particularly those targeted toward men who have sex with men (MSM) and sex workers [2–4]. A recent mathematical model predicted that disruption in HIV prevention, testing, and treatment services could lead to an acute increase in HIV incidence and HIV-related deaths over a period of up to 1 year in sub-Saharan Africa [5]. To our knowledge, no data are available that can evaluate the impact of COVID-19 restrictions and related interruptions in HIV prevention services on HIV acquisition risk among MSM and transgender women in sub-Saharan Africa. We assessed differences in HIV incidence among HIV-negative MSM and transgender women participating in a multicentre cohort study in Kisumu, Nairobi, and coastal Kenya.

## Methods

### Study setting and participants

The study was conducted at the Anza Mapema clinic in Kisumu [6], the Sex Workers Outreach Programme (SWOP) City clinic in Nairobi, the KEMRI clinics at Mtwapa and Malindi (both in coastal Kenya).

Criteria for study eligibility included: assigned to male gender at birth and identifying as male or transgender female, aged 18–29 years, resident of Kisumu, Nairobi or Coast, HIV-negative, willing to provide informed consent, and had anal intercourse with a man in the past 3 months. Volunteers were assessed for eligibility between September 2019 and May 2021.

In response to COVID-19 lockdown measures, all study sites were closed by 23 March 2020. Sites began to reopen as determined by local approval: 20 and 21 October 2020 (Nairobi, and Kisumu), and 9 November 2020 (Malindi). The Mtwapa clinic remained closed for administrative reasons; research procedures for Mtwapa participants finally resumed on 26 October 2021 at a nearby community-based organisation (HAPA Kenya, Nyali, Mombasa) [7]. During the lockdown period, participants had access to HIV prevention services through community-based programmes.

### Study procedures

Individuals were followed quarterly, with HIV counselling and testing at each study visit. At enrolment and all subsequent study visits, participants completed an audio computer-assisted self-interview (ACASI) in English, Swahili, or Dholuo for sociodemographics, sexual behaviours, intimate partner violence, depressive symptoms [Patient Health Questionnaire 9 (PHQ-9)], alcohol use [Alcohol Use Disorder Identification Test (AUDIT)], use of substances other than alcohol and tobacco [Drug Abuse Screening Test 10 (DAST-10)], childhood abuse, sexual stigma (abridged China MSM Stigma Scale) [8], intimate partner violence (IPV), social harms, perceived HIV risks, use of preexposure prophylaxis (PrEP), and use of feminizing hormones.

### Measures

#### Outcomes

*Follow-up period:* Follow-up was divided into two periods: during COVID-19-related clinic closure, defined as time from date of site closure until the first visit that a participant made following reopening of the study site, and after COVID-19-related clinic closure, defined as all visits that occurred during follow-up after the initial visit following reopening of the study site.

*Retained:* A participant was defined as retained in the cohort if they returned to the clinic following study resumption.

*HIV infection:* The estimated date of infection was calculated as described previously [9].

#### Covariates

Detailed information on covariates is provided in supplemental material. In short, data collected at enrolment included: age, gender identity, ever married to a woman, education, employment status, religion, and childhood abuse. We also obtained at each three-monthly visit: gender of last sexual partner (male or female), last male sexual partner category (regular, casual, paying or paid and partner numbers), condom use for receptive or insertive anal intercourse (RAI, IAI) (yes, no, no RAI, no IAI), and perceived risk of acquiring HIV (no chance at all to small chance, moderate chance to great chance).

The following were dichotomized (yes or no): RAI, IAI, receiving payment for sex, paying for sex, group sex, circumcision status, any intimate partner violence, other violence, PrEP use in the past 3 months, and AUDIT score at least 8.

The following information was collected at enrolment and/or half-yearly: moderate-to-severe depressive symptoms in the past 2 weeks (defined as having PHQ-9 score of 10–27), substance use disorder in past year (defined as having a DAST-10 score ≥3), childhood abuse and current use of feminizing hormones. Sexual stigma was collected at enrolment and half-yearly and measured using a continuous score ranging from 0 to 33.

### Statistical analysis

We compared demographic and behavioural characteristics at enrolment of participants who did versus those who did not return for follow-up after COVID-19-related clinic closure using Pearson's *χ*^2^ test for categorical variables and Mann–Whitney *U* test for continuous variables.

Follow-up time began at the date of site closure and continued until a positive HIV test result, loss to follow-up, withdrawal from study participation or 20 December 2022, whichever occurred first. Data collected on the first visit upon which the participant returned to the clinic after it reopened were assumed to represent behaviours during clinic closure. In all longitudinal analysis, certain information (e.g. PHQ-9) collected at baseline and half-yearly thereafter were carried forward to their subsequent quarterly visits.

Due to the few incident cases, we suspected that parameter estimates from standard regression techniques could be uncertain [10]. To minimize this bias, we used a penalized regression approach whereby uncertain estimates from the data are pulled towards more realistic ones with the use of prior distributions [11]. Therefore, to estimate the HIV incidence rate, incidence rate ratios (IRR) and the 95% credible intervals (CrI), we fitted a Poisson model using a Bayesian approach.

We calculated the proportion of individuals reporting any RAI, condomless RAI, any IAI, condomless IAI, PrEP use during the past 3 months and those with a PHQ-9 score of 10–27, AUDIT score at least 8 and DAST-10 score at least 3. We modelled these endpoints during clinic closure and after reopening. We calculated odds ratios (OR) and their 95% confidence intervals (CI) comparing the odds of having an endpoint for the two timepoints and tested for changes between timepoints using a Wald *χ*^2^ test.

The KEMRI Ethics Review Committee approved the study.

## Results

Most (70.8%) of the 794 cohort participants were 18–24 years old, and about 1 in 10 (12.8%) identified as transgender women. Overall, 690 (86.9%) returned to follow-up after clinic reopening (details in Supplemental Table 1). Compared with participants who were lost to follow-up, participants who returned were less likely to have been recruited from Mtwapa (*P* < 0.001), to be circumcised (*P* = 0.009), and to have reported IPV (*P* = 0.009), and more likely to have harmful or hazardous drinking by AUDIT score (*P* = 0.033) or to report a moderate-to-great perceived risk of HIV acquisition (*P* < 0.001).

Supplemental Table 2 presents cohort characteristics by site. One in seven (14%) participants had a PHQ-9 score of at least 10, indicating moderate-to-severe depressive symptoms. Over a third (37%) of participants had an AUDIT score of at least 8, suggesting hazardous alcohol use. Three in 10 (30%) participants reported a DAST-10 score of at least 3, indicating problematic substance use in the past year. Participants reported a median stigma score of 7 (IQR 3–13), and two-thirds (64%) reported any childhood abuse.

### Correlates of HIV acquisition

Overall, 690 participants with at least one follow-up visit contributed 1677.6 person-years of observation with a median of 32.7 months (IQR 30.5–34.9). There was a total of 664.3 person-years during clinic closure and 1013.3 person-years after clinic reopening. Twenty-four participants acquired HIV during follow-up, for an estimated HIV incidence rate of 1.38/100 person-years [95% CrI 0.92–1.97]. The HIV incidence rate was 2.05/100 person-years (95% CrI 1.22–3.26) during COVID-19 restrictions and 0.96/100 person-years (95% CrI 0.41–2.07) after COVID-19 restrictions (IRR 0.47, 95% CrI 0.20–1.01), a 43% reduction (Table 1).

**Table 1:** Risk factors for HIV acquisition among 690 Kenyan MSM and transgender women who returned for follow-up, March 2020 to December 2022.

Characteristics	HIV incident cases (*n* = 24)		Bivariable analysis	Multivariable analysis (*N* = 690)
	*n*/100 person-years	IR (95% CrI)^b^	IRR (95% CrI)	aIRR (95% CrI)
Follow-up period^a^				
During COVID-19 restrictions	14/664.3	2.05 (1.22–3.26)	Reference	Reference
After COVID-19 restrictions	10/1013.3	0.96 (0.41–2.07)	0.47 (0.20–1.01)	0.57 (0.23–1.33)
Study site				
Kisumu	2/703.7	0.62 (0.27–1.23)	Reference	Reference
Nairobi	10/617.1	1.57 (0.57–4.45)	2.56 (0.93–7.23)	2.20 (0.82–6.29)
Mtwapa	11/208.5	5.09 (1.93–13.56)	**8.28 (3.14–22.04)**	**5.40 (2.04–15.52)**
Malindi	1/148.3	0.47 (0.02–2.88)	0.77 (0.03–4.68)	0.70 (0.04–4.37)
Age group (years)				
18–24	11/833.7	1.36 (0.78–2.27)	Reference	
≥25	13/837.7	1.52 (0.68–3.22)	1.12 (0.50–2.37)	
Gender identity				
Male	22/1459.7	1.52 (1.00–2.24)	Reference	
Transgender woman/female/other	2/215.4	0.77 (0.10–2.68)	0.51 (0.07–1.76)	
Ever married to a female				
No	21/1480.4	1.44 (0.93–2.10)	Reference	
Yes	3/197.2	1.37 (0.29–3.40)	0.95 (0.20–2.77)	
Education				
Primary	7/256.2	2.52 (1.18–4.60)	Reference	
Secondary	13/990.5	1.27 (0.53–3.19)	0.50 (0.21–1.26)	
Higher/tertiary/other	4/430.9	0.86 (0.20–2.66)	0.34 (0.08–1.05)	
Employment				
Unemployed	8/675.9	1.26 (0.69–2.21)	Reference	
Employed	6/338.1	1.69 (0.57–4.33)	1.34 (0.45–3.42)	
Self-employed	4/412.9	0.88 (0.23–2.49)	0.69 (0.19–1.97)	
Casual/other	5/247.0	1.86 (0.59–5.00)	1.47 (0.47–3.96)	
Religion				
Muslim	4/305.1	1.43 (0.60–3.08)	Reference	
Christian	17/1063.4	1.54 (0.61–4.14)	1.08 (0.43–2.89)	
Other/none	3/309.1	0.87 (0.02–3.27)	0.61 (0.11–2.28)	
Gender of last sexual partner				
Male	23/1407.2	1.65 (1.11–2.38)	Reference	
Female	1/263.9	0.26 (0.01–1.41)	**0.16 (0.05–0.85)**	NI
Last male sexual partner category				
Regular	11/997.2	1.17 (0.66–1.93)	Reference	
Casual	8/296.8	2.56 (0.98–6.07)	2.18 (0.84–5.17)	
Paying/paid	5/369.1	1.26 (0.39–3.36)	1.07 (0.33–2.86)	
Number of male partners, past 3 months				
0–2	8/880.0	1.05 (0.53–1.79)	Reference	
3-4	10/426.9	2.24 (0.88–5.51)	2.13 (0.83–5.24)	
≥5	5/348.4	1.33 (0.41–4.05)	1.27 (0.39–3.85)	
Receptive anal intercourse, past 3 months				
No	5/802.7	0.82 (0.39–1.48)	Reference	Reference
Yes	19/865.6	2.16 (1.02–4.92)	**2.64 (1.24–6.02)**	1.94 (0.88–4.80)
Condom use for receptive anal intercourse (RAI), past 3 months				
No	6/147.0	3.80 (1.27–9.38)	2.11 (0.71–5.20)	NI
Yes	13/721.5	1.80 (1.06–2.89)	Reference	
No RAI	5/802.4	0.59 (0.17–1.49)	**0.32 (0.09–0.83)**	
Insertive anal intercourse, past 3 months				
No	3/357.6	1.13 (0.46–2.28)	Reference	
Yes	21/1314.1	1.56 (0.69–4.17)	1.39 (0.61–3.70)	
Condom use for insertive anal intercourse (IAI), past 3 months				
No	12/204.3	6.13 (2.69–14.36)	**6.13 (2.69–14.36)**	NI
Yes	9/1108.3	0.93 (0.48–1.60)	Reference	
No IAI	3/357.6	0.81 (0.16–2.70)	0.81 (0.16–2.70)	
Receiving payment for sex, past 3 months				
No	11/774.8	1.46 (0.81–2.42)	Reference	
Yes	13/892.0	1.42 (0.65–3.12)	0.97 (0.45–2.14)	
Paid for sex, past 3 months				
No	18/1069.3	1.72 (1.07–2.53)	Reference	
Yes	6/599.0	0.95 (0.33–2.23)	0.56 (0.19–1.30)	
Group sex, past 3 months				
No	20/1401.8	1.46 (0.94–2.16)	Reference	
Yes	4/266.2	1.38 (0.38–3.62)	0.94 (0.26–2.48)	
Circumcised				
No	2/115.9	1.45 (0.96–2.09)	1.00 (0.14–3.53)	
Yes	22/1555.4	1.45 (0.21–5.13)	Reference	
Self-reported PrEP use, since last visit				
No	20/1255.4	1.56 (0.68–3.94)	1.36 (0.59–3.42)	
Yes	4/414.2	1.15 (0.50–2.31)	Reference	
Current use of feminizing hormones^a^				
No	22/1461.2	1.54 (1.01–2.22)	Reference	
Yes	2/214.0	0.79 (0.11–2.73)	0.51 (0.07–1.77)	
Intimate partner violence, past 3 months				
No (0)	17/1369.6	1.29 (0.80–1.96)	Reference	
Yes (≥1)	5/302.5	2.22 (0.84–4.95)	1.72 (0.65–3.83)	
Other socio harms, past 3 months				
No (0)	22/1504.5	1.47 (0.96–2.15)	Reference	
Yes (≥1)	2/167.0	1.02 (0.01–3.57)	0.69 (0.10–2.42)	
Perceived risk of acquiring HIV				
No chance at all to small chance	9/1223.2	0.86 (0.49–1.45)	Reference	Reference
Moderate to great chance	15/445.6	3.25 (1.55–6.90)	**3.78 (1.80–8.02)**	**3.03 (1.40–6.24)**
Depressive symptoms (PHQ-9), past 2 weeks				
Minimal to mild (0–9)	19/1418.5	1.38 (0.89–2.05)	Reference	
Moderate to severe (10–27)	5/259.1	1.80 (0.57–4.39)	1.31 (0.41–3.19)	
Disordered alcohol use (AUDIT), past year				
Low (0–7)	18/1092.2	1.66 (1.05–2.48)	Reference	
Hazardous (8–40)	6/597.7	0.98 (0.35–2.29)	0.59 (0.21–1.37)	
Problematic substance use (DAST-10), past year				
No (0–2)	20/1137.3	1.76 (1.13–2.64)	Reference	
Yes (≥3)	4/540.3	0.68 (0.18–1.82)	0.39 (0.10–1.03)	
Sexual stigma score (0–33)	–	1.01 (0.96–1.06)		
Childhood abuse				
No (0)	7/613.3	1.26 (0.65–2.19)	Reference	
Yes (≥1)	17/1064.3	1.58 (0.74–3.42)	1.25 (0.59–2.70)	

NI, not included in the multivariable because of high autocorrelation. Bold values indicate variables whose 95% CrI of the HR did not cross 1. AUDIT, alcohol use disorder identification; CrI, credible intervals; DAST-10, Drug Abuse Screening Test 10; IR, incidence rate; IRR, incidence rate ratio; PHQ-9, Patient Health Questionnaire 9; PrEP, preexposure prophylaxis.

a During COVID-19 restrictions: between 24 March 2020 and the first visit after reopening of study sites (i.e. 19 October 2020 for Nairobi, 21 October 2020 for Kisumu, 9 November 2020 for Malindi, and 26 October 2021 for Mtwapa). After COVID-19 restrictions: all visits that occurred during follow-up after the initial visit following reopening of the study site.

b Calculated from the Bayesian Poisson regression model described in the Methods.

Fourteen participants acquired HIV during COVID-19 restrictions (details in Supplemental Table 3). Of note, HIV incidence in the period before COVID-19 was 0.99 [95% Crl (0.27–3.75)], based on only three incident cases during this period of cohort accrual.

HIV incidence varied across the four sites: 5.09/100 person-years (95% CrI 1.93–13.56) in Mtwapa, 1.57/100 person-years (95% CrI 0.57–4.45) in Nairobi, 0.62/100 person-years (95% CrI 0.27–1.23) in Kisumu and 0.47/100 person-years (95% CrI 0.02–2.88) in Malindi.

In multivariable analysis with adjustment for study site and other participant characteristics, HIV incidence was not different between the two COVID-19 periods (adjusted IRR 0.57, 95% CrI 0.23–1.33). HIV incidence was increased for Mtwapa participants (IRR 5.40, 95% CrI 2.04–15.52) and for participants reporting a moderate-to-great perceived risk of acquiring HIV (IRR 3.03, 95% CrI 1.40–6.24, Table 1).

### Changes in risk behaviour, pre-exposure prophylaxis use, mental health and substance use by follow-up period

Compared with the period after COVID-19 restrictions, the proportion of participants reporting RAI, IAI, condomless RAI and IAI, and hazardous alcohol use was higher during COVID-19-related clinic closures, especially in Nairobi and Kisumu (Table 2; i.e.75.6% of cohort; in Supplemental Table 2; change in proportion of participants reporting RAI per site in Supplementary Figure 1).

**Table 2:** Differences in reported risk behaviour, preexposure prophylaxis use, mental health, and substance use between follow-up periods, among 690 Kenyan MSM and transgender women who returned to the cohort after COVID-19 restrictions, March 2020–Decemeber 2022.

	RAI	Condomless RAI	IAI	Condomless IAI	PrEP use	PHQ-9^b^	AUDIT	DAST-10^b^
Measures	OR (95% CI), per analysed period							
Follow-up period^a^								
During COVID-19 restrictions	1.74 (1.37–2.20)	1.40 (0.97–2.01)	1.31 (1.00–1.71)	1.24 (0.90–1.70)	1.13 (0.85–1.50)	1.23 (0.89–1.70)	1.74 (1.34–2.26)	0.85 (0.65–1.11)
After COVID-19 restrictions	Reference	Reference	Reference	Reference	Reference	Reference	Reference	Reference

OR (95% CI) stratified by site and period								
Site								
Kisumu	2.30 (1.59–3.34)	1.86 (1.01–3.43)	1.48 (0.98–2.25)	1.50 (0.87–2.58)	1.12 (0.70–1.79)	0.77 (0.45–1.33)	1.71 (1.13–2.59)	1.21 (0.80–1.82)
Nairobi	1.56 (1.07–2.30)	1.24 (0.67–2.29)	1.50 (0.97–2.31)	1.22 (0.74–2.04)	1.29 (0.82–2.03)	1.55 (0.92–2.63)	1.86 (1.20–2.87)	0.68 (0.44–1.06)
Mtwapa	0.87 (0.41–1.83)	1.16 (0.50–2.71)	0.68 (0.30–1.53)	0.57 (0.26–1.23)	0.47 (0.21–1.04)	1.87 (0.64–5.41)	0.99 (0.47–2.10)	0.46 (0.20–1.06)
Malindi	1.34 (0.62–2.93)	0.37 (0.11–1.33)	0.80 (0.33–1.95)	1.06 (0.41–2.76)	1.55 (0.66–3.65)	1.04 (0.42–2.60)	2.31 (1.04–5.13)	1.31 (0.59–2.92)

AUDIT, Alcohol Use Disorder Identification; CI, confidence interval; DAST-10, Drug Abuse Screening; IAI, insertive anal intercourse; OR, odds ratio; PHQ-9, Patient Health Questionnaire 9; PrEP, preexposure prophylaxis; RAI, receptive anal intercourse.

a During COVID-19 restrictions: between 24 March 2020 and the first visit after reopening of study sites (i.e. 19 October 2020 for Nairobi, 21 October 2020 for Kisumu, 9 November 2020 for Malindi and 26 October 2021 for Mtwapa). After COVID-19 restrictions: all visits that occurred during follow-up after the initial visit following reopening of the study site.

b Forty-six percent of data collected every 6 months were carried forward to the subsequent quarterly return visit from before COVID visit.

## Discussion

Among MSM and transgender women followed up in Kenya, we assessed the impact of COVID-19-related clinic closures on HIV-acquisition risk. The overall HIV incidence was moderately high during COVID-19 restrictions and had an approximately 43% reduction after the clinics reopened. Although a minority (13%) of study participants did not return to follow-up, it is uncertain how lost participants impacted our HIV incidence estimate. Compared with the period after COVID-19 restrictions eased and clinics reopened, the proportion of participants reporting RAI, IAI, and condomless RAI, and IAI, and hazardous alcohol use was substantially higher during restrictions, in the majority (76.5%) of the cohort (Nairobi and Kisumu).

That HIV incidence was higher during clinic closure may be at least in part because of difficulty accessing services provided at the cohort sites, including distribution of condoms and lubricants, HIV counselling and testing, and PrEP, as well as antiretroviral therapy (ART) for individuals who test positive at initial screening or during follow-up. These services were lacking entirely in Mtwapa, the site that remained closed following lifting of the COVID-19 lockdown restrictions.

We found that the risk of HIV acquisition was two-fold higher among participants who reported moderate-to-great perceived risk of HIV than that among participants who reported no-to-small risk. This suggests that despite awareness of their HIV acquisition risk, these participants faced challenges in effectively using HIV prevention tools [12]. Although PrEP for HIV prevention was freely available to all participants, only one in four participants reported PrEP use at baseline and PrEP use remained low throughout the study period. Earlier, we reported that among participants receiving PrEP during 2017–2019, only 14.5% had sufficient tenofovir diphosphate in dried blood spots to confer protection [13]. Similarly, a more recent study of MSM in a PrEP program in Kisumu found that just 14.6% participants had protective TFV-levels in DBS samples [14].

Our study had several limitations. Firstly, 13% of study participants did not return to follow-up after COVID-19 restrictions, and it is uncertain how this impacted our incidence estimate. Secondly, during the COVID-19 restrictions when the study clinics remained closed, we could not collect research data; hence, it is uncertain how risk behaviours and psychosocial factors changed during this period. Lastly, we imputed certain measures collected at baseline and half-yearly thereafter based on the last observation carried forward, which is likely to have led to an underestimation of changes in these psychosocial factors during the clinic closure period.

In conclusion, in this large multicentre study of MSM and transgender women in Kenya, we documented a moderately high HIV incidence during COVID-19-related clinic closures when alcohol use and sexual risk behaviour was increased. HIV incidence reduced to a baseline estimate when clinics reopened. HIV incidence varied by location and was markedly higher in Mtwapa, where services were the most disrupted. Although a reduction in HIV incidence may be in keeping with an overall trend for reducing incidence in these key populations, measures to upscale and ensure uninterrupted HIV prevention and treatment services for MSM and transgender women should be prioritized.

## Acknowledgements

We thank staff at the four participating research sites for their support to the study, and study participants for their commitment to the study. Special thanks go to HAPA Kenya at Mombasa for enabling continuation of the study in Mombasa after the Mtwapa clinic was closed. The KEMRI HIV/Sexually Transmitted Infection (STI) Research group at KEMRI-Wellcome Trust is financially supported by the International AIDS vaccine Initiative (IAVI), which receives generous support of the American people through the United States Agency for International Development (USAID).

Authors’ contributions: E.J.S., F.O.O, J.K., R.C.B., and S.M.G. designed the research study. D.O., J.N., O.C., A.T., F.O., and K.R. collected the data, E.W. managed data. E.W. and A.B. analysed data, E.W., F.O.O., and J.K. wrote the original draft of the manuscript. A.B., S.D.M., S.M.G., and E.J.S. critically reviewed and edited the manuscript. All authors have read and provided feedback on manuscript drafts and have approved the final manuscript.

Tatu Pamoja (three site) study group authors: Oscar Chirro (KEMRI/Wellcome Trust Research Programme, Kilifi, Kenya) – study performance; Alexander Thiong’o (KEMRI/Wellcome Trust Research Programme, Kilifi, Kenya) – study performance; Fredick Ogada (KEMRI/Wellcome Trust Research Programme, Kilifi, Kenya) – study performance; Jennifer Kanungi (KEMRI/Wellcome Trust Research Programme, Kilifi, Kenya) – study performance; Maureen Akolo [Sex Worker Outreach Program (SWOP), Nairobi, Kenya and Partners for Health and Development in Africa (PHDA)] – study performance; Anthony Kariri [Sex Worker Outreach Program (SWOP), Nairobi, Kenya and Partners for Health and Development in Africa (PHDA)] – study performance and data management; Kelly Ramko (KEMRI/Wellcome Trust Research Programme, Kilifi, Kenya) – study performance and laboratory oversight; George Owino (International AIDS Vaccine Initiative) – study performance and stake holder engagement; Vincent Muturi-Kioi (International AIDS Vaccine Initiative) – study performance and stake holder engagement; Pat Fast (International AIDS Vaccine Initiative) – study performance and stake holder engagement.

Funding for the study was also provided through an Investigator Research Reward (IRR) to E.W. (IRR WHO-IIR01, IAVI), through a career development year award to E.W. (DELTAS, Wellcome Trust) and through the Sub-Saharan African Network for TB/HIV Research Excellence, a Developing Excellence in Leadership, Training and Science (DELTAS) Africa programme (Del-22–007) with support from Wellcome Trust and the UK Foreign, Commonwealth & Development Office and is part of the EDCPT2 programme supported by the European Union; the Bill & Melinda Gates Foundation (INV-033558); and Gilead Sciences Inc., (19275). The views expressed in this publication are those of the authors and not necessarily those of AAS, NEPAD Agency, Wellcome Trust, or the UK government. This research was funded in part by the Wellcome Trust. For the purpose of Open Access, the author has applied a CC-BY public copyright licence to any author accepted manuscript version arising from this submission. This manuscript was submitted for publication with permission from the Director of KEMRI.

Data sharing statement: A curated copy of the underlying data and analysis code has been deposited in the Harvard Dataverse repository (DOI) and may be requested from the KEMRI-Wellcome programme.

### Conflicts of interest

There are no conflicts of interest.

## Supplementary Material

Supplemental Digital Content

## Supplementary Material

Supplemental Digital Content
