# Supplementary material for: Impact of coronavirus disease 2019-related clinic closures on HIV incidence in young adult MSM and transgender women in Kenya
Source: AIDS. 2023 Nov 27;38(3):407–13. doi: 10.1097/QAD.0000000000003782 (PMC10842664; doi:10.1097/QAD.0000000000003782)
Supplement: Supplemental Digital Content [file aids-38-407-s001.docx]

**Supplemental Table 1. Characteristics at enrolment of HIV negative MSM & Transgender women in Kenya, Sep 2019 – Feb 2020**

| **Characteristics** | | **Overall**  **(n = 794)** | | **Returned**  **(n = 690)** | | **Did not return***  **(n = 104)** | | ***P* value** |
| --- | --- | --- | --- | --- | --- | --- | --- | --- |
|  |  | **n (%)** | | **n (%)** | | **n (%)** | |  |
| Study site | |  |  |  |  |  |  | **<0.001** |
|  | Kisumu | 300 | (37.8) | 279 | (40.4) | 21 | (20.2) |  |
|  | Nairobi | 297 | (37.4) | 249 | (36.1) | 48 | (46.2) |  |
|  | Mtwapa | 129 | (16.2) | 95 | (13.8) | 34 | (32.7) |  |
|  | Malindi | 68 | (8.6) | 67 | (9.7) | 1 | (1.0) |  |
| Age (years) | |  |  |  |  |  |  | 0.310 |
|  | 18-24 | 562 | (70.8) | 484 | (70.1) | 78 | (75.0) |  |
|  | ≥25 | 232 | (29.2) | 206 | (29.9) | 26 | (25.0) |  |
| Gender identity | |  |  |  |  |  |  | 0.910 |
|  | Male | 691 | (87.0) | 601 | (87.1) | 90 | (86.5) |  |
|  | Transgender woman/female/other^1,2^ | 102 | (12.8) | 88 | (12.8) | 14 | (13.5) |  |
| Ever married to a female | |  |  |  |  |  |  | 0.953 |
|  | No | 701 | (88.3) | 609 | (88.3) | 92 | (88.5) |  |
|  | Yes | 93 | (11.7) | 81 | (11.7) | 12 | (11.5) |  |
| Education | |  |  |  |  |  |  | 0.932 |
|  | Primary | 125 | (15.7) | 109 | (15.8) | 16 | (15.4) |  |
|  | Secondary | 468 | (58.9) | 405 | (58.7) | 63 | (60.6) |  |
|  | Higher/tertiary/other | 201 | (25.3) | 176 | (25.5) | 25 | (24.0) |  |
| Employment^1^ | |  |  |  |  |  |  | 0.500 |
|  | Unemployed | 315 | (39.7) | 281 | (40.7) | 34 | (32.7) |  |
|  | Employed | 156 | (19.6) | 135 | (19.6) | 21 | (20.2) |  |
|  | Self-employed | 193 | (24.3) | 165 | (23.9) | 28 | (26.9) |  |
|  | Casual/other | 128 | (16.1) | 107 | (15.5) | 21 | (20.2) |  |
| Religion | |  |  |  |  |  |  | 0.311 |
|  | Muslim | 149 | (18.8) | 125 | (18.1) | 24 | (23.1) |  |
|  | Christian | 495 | (62.3) | 437 | (63.3) | 58 | (55.8) |  |
|  | Other/none | 150 | (18.9) | 128 | (18.6) | 22 | (21.2) |  |
| Gender of last sexual partner^1^ | |  |  |  |  |  |  | 0.660 |
|  | Male | 691 | (87.0) | 603 | (87.4) | 88 | (84.6) |  |
|  | Female | 102 | (12.8) | 86 | (12.5) | 16 | (15.4) |  |
| Last male sexual partner category^1^ | |  |  |  |  |  |  | 0.334 |
|  | Regular | 406 | (51.1) | 348 | (50.4) | 58 | (55.8) |  |
|  | Casual | 138 | (17.4) | 126 | (18.3) | 12 | (11.5) |  |
|  | Paying/paid | 247 | (31.1) | 213 | (30.9) | 34 | (32.7) |  |
| Number of male partners, past 3 months^1^ | |  |  |  |  |  |  | 0.263 |
|  | 0-2 | 286 | (36.0) | 245 | (35.5) | 41 | (39.4) |  |
|  | 3-4 | 217 | (27.3) | 191 | (27.7) | 26 | (25.0) |  |
|  | ≥5 | 286 | (36.0) | 251 | (36.4) | 35 | (33.7) |  |
| Receptive anal intercourse (RAI), past 3 months^1^ | |  |  |  |  |  |  | 0.409 |
|  | No | 301 | (37.9) | 266 | (38.6) | 35 | (33.7) |  |
|  | Yes | 488 | (61.5) | 419 | (60.7) | 69 | (66.3) |  |
| Condom use for RAI, past 3 months^1^ | |  |  |  |  |  |  | 0.753 |
|  | No | 100 | (12.6) | 85 | (12.3) | 15 | (14.4) |  |
|  | Yes | 392 | (49.4) | 338 | (49.0) | 54 | (51.9) |  |
|  | No RAI | 301 | (37.9) | 266 | (38.6) | 35 | (33.7) |  |
| Insertive anal intercourse (IAI), past 3 months^1^ | |  |  |  |  |  |  | 0.192 |
|  | No | 152 | (19.1) | 126 | (18.3) | 26 | (25.0) |  |
|  | Yes | 637 | (80.2) | 559 | (81.0) | 78 | (75.0) |  |
| Condom use for IAI, past 3 months^1^ | |  |  |  |  |  |  | 0.321 |
|  | No | 122 | (15.4) | 110 | (15.9) | 12 | (11.5) |  |
|  | Yes | 519 | (65.4) | 453 | (65.7) | 66 | (63.5) |  |
|  | No IAI | 152 | (19.1) | 126 | (18.3) | 26 | (25.0) |  |
| Receiving payment for sex, past 3 months^1^ | |  |  |  |  |  |  | 0.646 |
|  | No | 316 | (39.8) | 272 | (39.4) | 44 | (42.3) |  |
|  | Yes | 474 | (59.7) | 414 | (60.0) | 60 | (57.7) |  |
| Paid for sex, past 3 months^1^ | |  |  |  |  |  |  | 0.654 |
|  | No | 498 | (62.7) | 436 | (63.2) | 62 | (59.6) |  |
|  | Yes | 294 | (37.0) | 252 | (36.5) | 42 | (40.4) |  |
| Group sex, past 3 months^1^ | |  |  |  |  |  |  | 0.663 |
|  | No | 641 | (80.7) | 560 | (81.2) | 81 | (77.9) |  |
|  | Yes | 152 | (19.1) | 129 | (18.7) | 23 | (22.1) |  |
| Circumcised^1^ | |  |  |  |  |  |  | **0.009** |
|  | No | 43 | (5.4) | 41 | (5.9) | 2 | (1.9) |  |
|  | Yes | 750 | (94.5) | 649 | (94.1) | 101 | (97.1) |  |
| Self-reported PrEP use^1^ | |  |  |  |  |  |  | 0.527 |
|  | No | 575 | (72.4) | 502 | (72.8) | 73 | (70.2) |  |
|  | Yes | 216 | (27.2) | 186 | (27.0) | 30 | (28.8) |  |
| Current use of feminizing hormones^1^ | |  |  |  |  |  |  | 0.310 |
|  | No | 699 | (88.0) | 612 | (88.7) | 87 | (83.7) |  |
|  | Yes | 86 | (10.8) | 71 | (10.3) | 15 | (14.4) |  |
| Intimate partner violence, past 3 months | |  |  |  |  |  |  | **0.009** |
|  | No (0) | 646 | (81.4) | 571 | (82.8) | 75 | (72.1) |  |
|  | Yes (≥1) | 148 | (18.6) | 119 | (17.2) | 29 | (27.9) |  |
| Other socio harms, past 3 months | |  |  |  |  |  |  | 0.949 |
|  | No (0) | 719 | (90.6) | 625 | (90.6) | 94 | (90.4) |  |
|  | Yes (≥1) | 75 | (9.4) | 65 | (9.4) | 10 | (9.6) |  |
| Perceived risk of acquiring HIV^1^ | |  |  |  |  |  |  | **<0.001** |
|  | No chance at all to small chance | 564 | (71.0) | 484 | (70.1) | 80 | (76.9) |  |
|  | Moderate to great chance | 228 | (28.7) | 206 | (29.9) | 22 | (21.2) |  |
| Depressive symptoms (PHQ-9), past 2 weeks | |  |  |  |  |  |  | 0.334 |
|  | Minimal to mild (0-9) | 686 | (86.4) | 593 | (85.9) | 93 | (89.4) |  |
|  | Moderate to severe (10-27) | 108 | (13.6) | 97 | (14.1) | 11 | (10.6) |  |
| Disordered alcohol use (AUDIT), past year | |  |  |  |  |  |  | **0.033** |
|  | Low (0-7) | 514 | (64.7) | 437 | (63.3) | 77 | (74.0) |  |
|  | Hazardous (8-40) | 280 | (35.3) | 253 | (36.7) | 27 | (26.0) |  |
| Problematic substance use (DAST-10), past year | |  |  |  |  |  |  | 0.873 |
|  | No (0-2) | 555 | (69.9) | 483 | (70.0) | 72 | (69.2) |  |
|  | Yes (≥3) | 239 | (30.1) | 207 | (30.0) | 32 | (30.8) |  |
| Sexual stigma score (0-33) [Median (IQR)] | | 7 | (3-13) | 7 | (3-13) | 7 | (3-11) | 0.567 |
| Childhood abuse | |  |  |  |  |  |  | 0.328 |
|  | No (0) | 294 | (37.0) | 251 | (36.4) | 43 | (41.3) |  |
|  | Yes (≥1) | 500 | (63.0) | 439 | (63.6) | 61 | (58.7) |  |

PHQ-9=Patient Health Questionnaire 9, AUDIT=Alcohol Use Disorder Identification, DAST-10=Drug Abuse Screening Test 10, PrEP=pre-exposure prophylaxis, IQR=Interquartile range

^1^Missing 1 value for gender identity, gender of last sex partner, group sex, condom use for RAI, IAI and circumcised, 2 values for employment, paid for sex and HIV risks, 3 values for last sexual partner category and self-reported PrEP use, 4 values for received payment for sex, 5 values for number of male partners, RAI and IAI, 9 values for gender affirming therapy.

^2^Other gender identity n=8

*After COVID-19 restrictions ended.

**Supplemental Table 2. Characteristics at enrolment of Kenyan HIV-negative MSM and transgender women who returned to the center after COVID-19 restrictions, Mar 2020 – Dec 2022**

| **Characteristics** | | **Overall**  **(n=690)** | |  | **Kisumu**  **(n=279)** | |  | **Nairobi (n=249)** | |  | **Mtwapa**  **(n=95)** | |  | **Malindi (n=67)** | |
| --- | --- | --- | --- | --- | --- | --- | --- | --- | --- | --- | --- | --- | --- | --- | --- |
|  |  | **n (%)** | |  | **n (%)** | |  | **n (%)** | |  | **n (%)** | |  | **n (%)** | |
| Age (years) | |  |  |  |  |  |  |  |  |  |  |  |  |  |  |
|  | 18-24 | 484 | (70.1) |  | 185 | (66.3) |  | 206 | (82.7) |  | 56 | (58.9) |  | 37 | (55.2) |
|  | ≥25 | 206 | (29.9) |  | 94 | (33.7) |  | 43 | (17.3) |  | 39 | (41.1) |  | 30 | (44.8) |
| Gender identity | |  |  |  |  |  |  |  |  |  |  |  |  |  |  |
|  | Male | 601 | (87.1) |  | 240 | (86.0) |  | 232 | (93.2) |  | 84 | (88.4) |  | 45 | (67.2) |
|  | Transgender woman/female/other^1,2^ | 88 | (12.8) |  | 39 | (14.0) |  | 17 | (6.8) |  | 11 | (11.6) |  | 21 | (31.3) |
| Ever married to a female | |  |  |  |  |  |  |  |  |  |  |  |  |  |  |
|  | No | 609 | (88.3) |  | 242 | (86.7) |  | 231 | (92.8) |  | 81 | (85.3) |  | 55 | (82.1) |
|  | Yes | 81 | (11.7) |  | 37 | (13.3) |  | 18 | (7.2) |  | 14 | (14.7) |  | 12 | (17.9) |
| Education | |  |  |  |  |  |  |  |  |  |  |  |  |  |  |
|  | Primary | 109 | (15.8) |  | 39 | (14.0) |  | 23 | (9.2) |  | 22 | (23.2) |  | 25 | (37.3) |
|  | Secondary | 405 | (58.7) |  | 170 | (60.9) |  | 152 | (61.0) |  | 45 | (47.4) |  | 38 | (56.7) |
|  | Higher/tertiary/other | 176 | (25.5) |  | 70 | (25.1) |  | 74 | (29.7) |  | 28 | (29.5) |  | 4 | (6.0) |
| Employment^1^ | |  |  |  |  |  |  |  |  |  |  |  |  |  |  |
|  | Unemployed | 281 | (40.7) |  | 91 | (32.6) |  | 124 | (49.8) |  | 36 | (37.9) |  | 30 | (44.8) |
|  | Employed | 135 | (19.6) |  | 58 | (20.8) |  | 46 | (18.5) |  | 23 | (24.2) |  | 8 | (11.9) |
|  | Self-employed | 165 | (23.9) |  | 89 | (31.9) |  | 47 | (18.9) |  | 14 | (14.7) |  | 15 | (22.4) |
|  | Casual/other | 107 | (15.5) |  | 40 | (14.3) |  | 32 | (12.9) |  | 21 | (22.1) |  | 14 | (20.9) |
| Religion | |  |  |  |  |  |  |  |  |  |  |  |  |  |  |
|  | Muslim | 125 | (18.1) |  | 52 | (18.6) |  | 23 | (9.2) |  | 26 | (27.4) |  | 24 | (35.8) |
|  | Christian | 437 | (63.3) |  | 176 | (63.1) |  | 173 | (69.5) |  | 56 | (58.9) |  | 32 | (47.8) |
|  | Other/none | 128 | (18.6) |  | 51 | (18.3) |  | 53 | (21.3) |  | 13 | (13.7) |  | 11 | (16.4) |
| Gender of last sexual partner^1^ | | |  |  |  |  |  |  |  |  |  |  |  |  |  |
|  | Male | 603 | (87.4) |  | 243 | (87.1) |  | 230 | (92.4) |  | 76 | (80.0) |  | 54 | (80.6) |
|  | Female | 86 | (12.5) |  | 36 | (12.9) |  | 18 | (7.2) |  | 19 | (20.0) |  | 13 | (19.4) |
| Last male sexual partner category^1^ | | | |  |  |  |  |  |  |  |  |  |  |  |  |
|  | Regular | 348 | (50.4) |  | 165 | (59.1) |  | 130 | (52.2) |  | 30 | (31.6) |  | 23 | (34.3) |
|  | Casual | 126 | (18.3) |  | 43 | (15.4) |  | 41 | (16.5) |  | 27 | (28.4) |  | 15 | (22.4) |
|  | Paying/paid | 213 | (30.9) |  | 69 | (24.7) |  | 77 | (30.9) |  | 38 | (40.0) |  | 29 | (43.3) |
| Number of male partners, past 3 months^1^ | |  |  |  |  |  |  |  |  |  |  |  |  |  |  |
|  | 0-2 | 245 | (35.5) |  | 122 | (43.7) |  | 86 | (34.5) |  | 15 | (15.8) |  | 22 | (32.8) |
|  | 3-4 | 191 | (27.7) |  | 88 | (31.5) |  | 61 | (24.5) |  | 25 | (26.3) |  | 17 | (25.4) |
|  | ≥5 | 251 | (36.4) |  | 68 | (24.4) |  | 101 | (40.6) |  | 54 | (56.8) |  | 28 | (41.8) |
| Receptive anal intercourse (RAI), past 3 months^1^ | |  |  |  |  |  |  |  |  |  |  |  |  |  |  |
|  | No | 266 | (38.6) |  | 95 | (34.1) |  | 111 | (44.6) |  | 36 | (37.9) |  | 24 | (35.8) |
|  | Yes | 419 | (60.7) |  | 183 | (65.6) |  | 138 | (55.4) |  | 56 | (58.9) |  | 42 | (62.7) |
| Condom use for RAI, past 3 months^1^ | |  |  |  |  |  |  |  |  |  |  |  |  |  |  |
|  | No | 85 | (12.3) |  | 35 | (12.5) |  | 21 | (8.4) |  | 17 | (17.9) |  | 12 | (17.9) |
|  | Yes | 338 | (49.0) |  | 149 | (53.4) |  | 117 | (47.0) |  | 42 | (44.2) |  | 30 | (44.8) |
|  | No RAI | 266 | (38.6) |  | 95 | (34.1) |  | 111 | (44.6) |  | 36 | (37.9) |  | 24 | (35.8) |
| Insertive anal intercourse (IAI), past 3 months^1^ | |  |  |  |  |  |  |  |  |  |  |  |  |  |  |
|  | No | 126 | (18.3) |  | 51 | (18.3) |  | 48 | (19.3) |  | 20 | (21.1) |  | 7 | (10.4) |
|  | Yes | 559 | (81.0) |  | 227 | (81.4) |  | 200 | (80.3) |  | 72 | (75.8) |  | 60 | (89.6) |
| Condom use for IAI, past 3 months^1^ | |  |  |  |  |  |  |  |  |  |  |  |  |  |  |
|  | No | 110 | (15.9) |  | 37 | (13.3) |  | 34 | (13.7) |  | 22 | (23.2) |  | 17 | (25.4) |
|  | Yes | 453 | (65.7) |  | 191 | (68.5) |  | 167 | (67.1) |  | 53 | (55.8) |  | 42 | (62.7) |
|  | No IAI | 126 | (18.3) |  | 51 | (18.3) |  | 48 | (19.3) |  | 20 | (21.1) |  | 7 | (10.4) |
| Receiving payment for sex, past 3 months^1^ | |  |  |  |  |  |  |  |  |  |  |  |  |  |  |
|  | No | 272 | (39.4) |  | 126 | (45.2) |  | 100 | (40.2) |  | 31 | (32.6) |  | 15 | (22.4) |
|  | Yes | 414 | (60.0) |  | 150 | (53.8) |  | 149 | (59.8) |  | 64 | (67.4) |  | 51 | (76.1) |
| Paid for sex, past 3 months^1^ | |  |  |  |  |  |  |  |  |  |  |  |  |  |  |
|  | No | 436 | (63.2) |  | 176 | (63.1) |  | 154 | (61.8) |  | 73 | (76.8) |  | 33 | (49.3) |
|  | Yes | 252 | (36.5) |  | 102 | (36.6) |  | 95 | (38.2) |  | 22 | (23.2) |  | 33 | (49.3) |
| Group sex, past 3 months^1^ | |  |  |  |  |  |  |  |  |  |  |  |  |  |  |
|  | No | 560 | (81.2) |  | 226 | (81.0) |  | 215 | (86.3) |  | 74 | (77.9) |  | 45 | (67.2) |
|  | Yes | 129 | (18.7) |  | 52 | (18.6) |  | 34 | (13.7) |  | 21 | (22.1) |  | 22 | (32.8) |
| Circumcised | |  |  |  |  |  |  |  |  |  |  |  |  |  |  |
|  | No | 41 | (5.9) |  | 26 | (9.3) |  | 6 | (2.4) |  | 7 | (7.4) |  | 2 | (3.0) |
|  | Yes | 649 | (94.1) |  | 253 | (90.7) |  | 243 | (97.6) |  | 88 | (92.6) |  | 65 | (97.0) |
| Intimate partner violence, past 3 months | |  |  |  |  |  |  |  |  |  |  |  |  |  |  |
|  | No (0) | 571 | (82.8) |  | 231 | (82.8) |  | 208 | (83.5) |  | 78 | (82.1) |  | 54 | (80.6) |
|  | Yes (≥1) | 119 | (17.2) |  | 48 | (17.2) |  | 41 | (16.5) |  | 17 | (17.9) |  | 13 | (19.4) |
| Other socio harms, past 3 months | |  |  |  |  |  |  |  |  |  |  |  |  |  |  |
|  | No (0) | 625 | (90.6) |  | 249 | (89.2) |  | 230 | (92.4) |  | 82 | (86.3) |  | 64 | (95.5) |
|  | Yes (≥1) | 65 | (9.4) |  | 30 | (10.8) |  | 19 | (7.6) |  | 13 | (13.7) |  | 3 | (4.5) |
| Perceived risk of acquiring HIV | |  |  |  |  |  |  |  |  |  |  |  |  |  |  |
|  | No chance at all to small chance | 484 | (70.1) |  | 205 | (73.5) |  | 184 | (73.9) |  | 58 | (61.1) |  | 37 | (55.2) |
|  | Moderate to great chance | 206 | (29.9) |  | 74 | (26.5) |  | 65 | (26.1) |  | 37 | (38.9) |  | 30 | (44.8) |
| Depressive symptoms (PHQ-9), past 2 weeks | |  |  |  |  |  |  |  |  |  |  |  |  |  |  |
|  | Minimal to mild (0-9) | 593 | (85.9) |  | 244 | (87.5) |  | 211 | (84.7) |  | 81 | (85.3) |  | 57 | (85.1) |
|  | Moderate to severe (10-27) | 97 | (14.1) |  | 35 | (12.5) |  | 38 | (15.3) |  | 14 | (14.7) |  | 10 | (14.9) |
| Disordered alcohol use (AUDIT), past year | |  |  |  |  |  |  |  |  |  |  |  |  |  |  |
|  | Low (0-7) | 437 | (63.3) |  | 156 | (55.9) |  | 176 | (70.7) |  | 68 | (71.6) |  | 37 | (55.2) |
|  | Hazardous (8-40) | 253 | (36.7) |  | 123 | (44.1) |  | 73 | (29.3) |  | 27 | (28.4) |  | 30 | (44.8) |
| Problematic substance use (DAST-10), past year | |  |  |  |  |  |  |  |  |  |  |  |  |  |  |
|  | No (0-2) | 483 | (70.0) |  | 185 | (66.3) |  | 181 | (72.7) |  | 70 | (73.7) |  | 47 | (70.1) |
|  | Yes (≥3) | 207 | (30.0) |  | 94 | (33.7) |  | 94 | (37.8) |  | 25 | (26.3) |  | 20 | (29.9) |
| Sexual stigma score (0-33) [Median (IQR)] | | 7 | (3-13) |  | 6 | (1-12) |  | 9 | (4-14) |  | 6 | (3-11) |  | 9 | (4-17) |
| Childhood abuse | |  |  |  |  |  |  |  |  |  |  |  |  |  |  |
|  | No (0) | 251 | (36.4) |  | 96 | (34.4) |  | 94 | (37.8) |  | 35 | (36.8) |  | 26 | (38.8) |
|  | Yes (≥1) | 439 | (63.6) |  | 183 | (65.6) |  | 155 | (62.2) |  | 60 | (63.2) |  | 41 | (61.2) |
| Self-reported PrEP use^1^ | |  |  |  |  |  |  |  |  |  |  |  |  |  |  |
|  | No | 502 | (72.8) |  | 225 | (80.6) |  | 193 | (77.5) |  | 45 | (47.4) |  | 39 | (58.2) |
|  | Yes | 186 | (27.0) |  | 54 | (19.4) |  | 56 | (22.5) |  | 49 | (51.6) |  | 27 | (40.3) |
| Current use of feminizing hormones^1^ | |  |  |  |  |  |  |  |  |  |  |  |  |  |  |
|  | No | 612 | (88.7) |  | 242 | (86.7) |  | 226 | (90.8) |  | 88 | (92.6) |  | 56 | (83.6) |
|  | Yes | 71 | (10.3) |  | 34 | (12.2) |  | 22 | (8.8) |  | 4 | (4.2) |  | 11 | (16.4) |

PHQ-9=Patient Health Questionnaire 9, AUDIT=Alcohol Use Disorder Identification, DAST-10=Drug Abuse Screening Test 10, PrEP=pre-exposure prophylaxis, IQR=Interquartile range

^1^Missing 1 value for gender identity, gender of last sex partner, group sex, condom use for RAI, and IAI, 2 values for employment, paid for sex and self-reported PrEP use, 3 values for last sexual partner category and number of male partners, 4 values for received payment for sex, 5 values for RAI and IAI, 7 values for gender affirming therapy.

^2^Other gender identity n=8.

**F**

**E**

**D**

**C**

**A**

**B**

**H**

**G**

**Supplemental Figure 1. Proportion for (A) receptive anal intercourse (RAI), (B) condomless RAI, (C) insertive anal intercourse (IAI), (D) condomless RAI, (E) Pre-exposure prophylaxis (PrEP) use, (F) Elevated depressive symptoms (PHQ-9) past 2 weeks, (G) Elevated AUDIT, (H) Elevated DAST-10 (Drug Abuse Screening) in the past 3 months among 690 Kenyan MSM and Transgender women, between Oct 2020 and Dec 2022.** PHQ-9=Patient Health Questionnaire 9, AUDIT=Alcohol Use Disorder Identification.

*During COVID-19 restrictions: Between 24 March 2020 and the first visit after reopening of study sites (i.e., 19 October 2020 for Nairobi, 21 October 2020 for Kisumu, 9 November 2020 for Malindi and 26 October 2021 for Mtwapa). After COVID-19 restrictions: All visits that occurred during follow-up after the initial visit following reopening of the study site.

**Supplemental Table 3. HIV acquisition among Kenyan MSM & TGW, Mar 2020 – Dec 2022**

| **Number** | **Study site** | **Date of study site closure** | **Last documented HIV negative date** | **Documented date of diagnosis^1^** | **Estimated date of infection^2^** | **Date site reopened** |
| --- | --- | --- | --- | --- | --- | --- |
| 1 | Nairobi | 20-Mar-20 | 24-Sep-20 | 11-Nov-20 | 18-Oct-20 | 19-Oct-20 |
| 2 | Nairobi | 20-Mar-20 | 17-Feb-20 | 19-Nov-20 | 04-Jul-20 | 19-Oct-20 |
| 3 | Nairobi | 20-Mar-20 | 06-Feb-20 | 23-Nov-20 | 30-Jun-20 | 19-Oct-20 |
| 4 | Nairobi | 20-Mar-20 | 27-Jan-20 | 24-Nov-20 | 26-Jun-20 | 19-Oct-20 |
| 5 | Kisumu | 23-Mar-20 | 12-Feb-20 | 07-Aug-20 | 10-May-20 | 21-Oct-20 |
| 6 | Kisumu | 23-Mar-20 | 10-Jan-20 | 23-Feb-21 | 02-Aug-20 | 21-Oct-20 |
| 7 | Mtwapa | 18-Mar-20 | 09-Jan-20 | 26-Jul-21 | 17-Oct-20 | 26-Oct-21 |
| 8 | Mtwapa | 18-Mar-20 | 30-Jan-20 | 26-Oct-21 | 12-Dec-20 | 26-Oct-21 |
| 9 | Mtwapa | 18-Mar-20 | 15-Jan-20 | 27-Oct-21 | 05-Dec-20 | 26-Oct-21 |
| 10 | Mtwapa | 18-Mar-20 | 13-Jan-20 | 28-Oct-21 | 05-Dec-20 | 26-Oct-21 |
| 11 | Mtwapa | 18-Mar-20 | 21-Jan-20 | 03-Nov-21 | 12-Dec-20 | 26-Oct-21 |
| 12 | Mtwapa | 18-Mar-20 | 29-Jun-21 | 10-Nov-21 | 04-Sep-21 | 26-Oct-21 |
| 13 | Mtwapa | 18-Mar-20 | 29-Jan-20 | 15-Nov-21 | 22-Dec-20 | 26-Oct-21 |
| 14 | Mtwapa | 18-Mar-20 | 03-Dec-19 | 16-Nov-21 | 24-Nov-20 | 26-Oct-21 |

^1^First visit after COVID-19 restrictions.

^2^The estimated date of infection was calculated as 10 days before the sample collection date (if the sample tested positive for HIV RNA and negative for HIV serology) or the mid-point between the dates of the previously documented negative and subsequently positive HIV serologic test (if the sample with HIV positive serology tested negative for HIV RNA or if HIV RNA testing was not performed or 18 days before discordant rapid HIV antibody tests (with or without a positive HIV RNA result).
