# Supplementary material for: Impact of coronavirus disease 2019-related clinic closures on HIV incidence in young adult MSM and transgender women in Kenya
Source: AIDS. 2023 Nov 27;38(3):407–13. doi: 10.1097/QAD.0000000000003782 (PMC10842664; doi:10.1097/QAD.0000000000003782)
Supplement: Supplemental Digital Content [file aids-38-407-s002.docx]

Detailed study procedures

At the enrolment visit, contact information was collected and a biometric iris scan obtained for registration and linkage to a unique participant study ID. Individuals were then followed at study visits occurring every 3 months. At these follow-up visits, participants received an iris scan to verify their identity and their contact information were updated. A cloud-based database containing the iris scans of all participants allowed cross-checking individuals who may have participated in the study at one of the three other locations.

At enrollment and all subsequent study visits, participants completed an audio computer-assisted self-interview (ACASI) in English, Swahili or Dholuo for socio-demographics, sexual behaviors, intimate partner violence, depressive symptoms [Patient Health Questionnaire 9 (PHQ-9)], alcohol use [Alcohol Use Disorder Identification Test (AUDIT)], use of substances other than alcohol and tobacco [Drug Abuse Screening Test 10 (DAST-10)], childhood abuse, sexual stigma (abridged China MSM Stigma Scale) (13), intimate partner violence (IPV), social harms, perceived HIV risks, use of pre-exposure prophylaxis (PrEP), and use of feminizing hormones.

In addition, counselling and testing for HIV using rapid antibody tests, collection of a 4-mL blood sample for storage, symptom directed medical history and physical examination were performed at all study visits. All participants were evaluated for symptoms of acute HIV infection (AHI). When symptoms suggestive of AHI or sexually transmitted infection (STI) were reported and rapid tests were negative or discordant, an Xpert® HIV Qual assay was used to detect AHI. Any study participant who acquired HIV during follow up was linked to existing care programmes at each site or at the clinic of their choice. All participants were assessed for PrEP eligibility using both Kenyan Ministry of Health (MoH) criteria and the empiric algorithm for PrEP targeting in MSM (14, 15), and those not yet on PrEP were encouraged to start. Participants eligible for PrEP and interested in taking it were provided with a PrEP supply for 90-day daily use. Participants were treated for symptomatic STI and provided with hepatitis B virus vaccination as recommended by the MoH. Participants received 650 Kenyan shillings (US$5.6) for scheduled follow-up study visits.

Measures

Outcomes

*Follow-up period:* Follow-up was divided into two periods: (1) during COVID-19 restrictions, defined as time from date of site closure until the first visit that a participant made following reopening of the study site, and (2) after COVID-19 restrictions, defined as all visits that occurred during follow-up after the initial visit following reopening of the study site.

*Retained*: A participant was defined as retained in the cohort if they returned to the clinic following study resumption at their clinical site.

*HIV infection:* The estimated date of infection was calculated as 10 days before the sample collection date (if the sample tested positive for HIV RNA and negative for HIV serology) or the mid-point between the dates of the previously documented negative and subsequently positive HIV serologic test (if the sample with HIV positive serology tested negative for HIV RNA or if HIV RNA testing was not performed) (16).

**Covariates**

Data collected at enrolment included: age, gender identity, ever married to a woman, education, employment status, religion, and childhood abuse (defined as having experienced any violence, mistreatment, or abuse during childhood). We also obtained the following information at enrolment and at each 3-monthly study visit: gender of last sexual partner (male or female), last male sexual partner category (regular, casual, paying or paid), number of sexual partners (0, 1, and >1), number of male sexual partners (0, 1, and >1), condom use for receptive anal intercourse (RAI) (yes, no, no RAI), condom use for insertive anal intercourse (IAI) (yes, no, no IAI) and perceived risk of acquiring HIV (no chance at all to small chance, moderate chance to great chance).

The following were dichotomized (yes or no): RAI, IAI, receiving payment for sex, paying for sex, group sex, circumcision status, any intimate partner violence (defined as being in a relationship with a person in the past 3 months who threatens, frightens, insults, or treats the participant badly or physically hurts or forces the participant to participate in sexual activities that were uncomfortable), other violence (defined as having been physically assaulted or raped or forced to have sex in the past 3 months), PrEP use in the past 3 months, and alcohol use disorder in past year (defined as having an AUDIT score ≥8). The following information was collected at enrolment and/ or half-yearly: moderate to severe depressive symptoms in the past 2 weeks (defined as having PHQ-9 score of 10-27), substance use disorder in past year (defined as having a DAST-10 score ≥3), childhood abuse and current use of feminizing hormones. Sexual stigma was collected at enrolment and half-yearly and measured using a continuous score ranging from 0-33, with higher scores reflecting more sexual stigma.

Statistical analysis

We compared demographic and behavioral characteristics at enrollment of participants who did versus those who did not return for follow-up after COVID-19 restrictions using Pearson’s χ^2^ test for categorical variables and Mann-Whitney U test for continuous variables. For individuals who returned for follow-up, we also compared their characteristics at enrolment across study sites.

Follow-up time began at the date of site closure and continued until positive HIV testing (through rapid or RNA test), loss to follow-up, withdrawal of study participation or December 20, 2022, whichever occurred first. Data collected on the first visit upon which the participant returned to the clinic after it reopened were assumed to represent behaviours during the COVID-19 pandemic. In all longitudinal analysis, certain information (i.e., DAST-10, PHQ-9, sexual stigma score and gender affirming hormones) collected at baseline and half-yearly thereafter were carried forward to their subsequent quarterly visits.

Due to the few incident cases that were expected, we suspected that parameter estimates from standard regression techniques could be exaggerated and uncertain (17). To minimize this bias, we used a penalized regression approach whereby uncertain estimates from the data are pulled towards more realistic one with the use of prior distributions (18). Therefore, to estimate the HIV incidence rate (IR), incidence rate ratios (IRR) and the 95% credible intervals (CrI), we fitted a Poisson model using a Bayesian approach. A weakly-informative prior for each determinant was specified as a log-normal distribution with mean 0 and variance 0.5. An informative prior of the intercept was also specified as a normal distribution with mean 0.02 and variance 0.5. Using these priors together with the data, a posterior distribution of the IRRs was estimated with Markov Chain Monte Carlo methods from the “bayes” family of commands in STATA. The median of this distribution defined the parameter estimate (i.e., posterior-IRR) and the 2.5% and 97.5% quantiles defined the 95% credible interval (CrI). Variables whose 95% CrI of the IRR did not cross 1 in the bivariable analysis were included along with site and follow-up period in a full multivariable model. After removing covariates that did not achieve convergence (i.e., Gelman-Rubin statistic not less than 1.1) or had high autocorrelation, we arrived at a final multivariable model.

We calculated the proportion of individuals reporting: (a) any RAI, (b) condomless RAI, (c) any IAI, (d) condomless IAI, (e) PrEP use during the past 3 months, (f) PHQ-9 score of 10-27, (g) AUDIT score ≥8 and (h) DAST-10 score ≥3. We modelled these endpoints at distinct timepoints during follow-up (during and after COVID-19 restriction) using a mixed effect logistic regression model with a random intercept for individual to account for between-participant variability. We calculated odds ratios (OR) and their 95% confidence intervals (CI) comparing the odds of having an endpoint for the two timepoints and tested for changes between timepoints using a Wald χ^2^ test. To assess whether changes in OR over the two follow-up visits differed between sites, we included an interaction term between follow-up time and site in the model. We calculated stratum-specific OR directly from this model.
